# Statins as Antifungal Agents: A Review on Drug Repurposing and Nanotechnology‐Driven Delivery Strategies

**DOI:** 10.1111/fcp.70046

**Published:** 2025-09-10

**Authors:** Dominique Mesquita e Silva, Laís de Souza Lacerda, Andrea de Souza Andrioli, Wilson Rodrigues Braz, Lara Melo Campos, Mayara Rodrigues Brandão de Paiva, Frederico Pittella, Rodrigo Luiz Fabri, Guilherme Diniz Tavares

**Affiliations:** ^1^ Postgraduate Program in Pharmaceutical Science Federal University of Juiz de Fora Juiz de Fora Minas Gerais Brazil; ^2^ Department of Pharmaceutical Science, Faculty of Pharmacy Federal University of Juiz de Fora Juiz de Fora Minas Gerais Brazil; ^3^ Department of Biochemistry, Institute of Biological Sciences Federal University of Juiz de Fora Juiz de Fora Minas Gerais Brazil

**Keywords:** antifungal activity, *Candida albicans*, candidiasis, drug repurposing, nanotechnology, statins

## Abstract

This review highlights the integration of drug repurposing and nanotechnology‐driven delivery strategies as innovative approaches to enhance the antifungal activity of statins against mucosal candidiasis, providing a framework for future translational research and clinical application. The rising prevalence of antifungal resistance and virulence factors of *Candida albicans* underscore the limitations of current therapies. Statins, commonly used as lipid‐lowering agents, have emerged as attractive repurposed drug candidates due to their ability to interfere with fungal ergosterol biosynthesis and Ras‐mediated signaling pathways. However, repurposed statins face major translational barriers, including poor water solubility, limited mucosal bioavailability, and dose‐dependent systemic toxicity. Nanotechnology‐driven delivery platforms offer versatile solutions to these challenges, enabling site‐directed delivery, improved stability, enhanced permeability, and controlled release. Lipid and polymeric nanocarriers, particularly chitosan‐based nanoparticles, enable controlled release and prolonged mucosal retention, making them suitable for localized antifungal therapy. This review explores the integration of statin repurposing with advanced drug delivery strategies as a novel therapeutic paradigm for mucosal candidiasis. Updated evidence demonstrating the antifungal potential of nano‐formulated statins is summarized, in conjunction with a general overview of design aspects relevant to optimizing delivery systems. Although still in early stages of investigation, this synergistic approach holds promise for overcoming resistance mechanisms and reducing the recurrence rates associated with existing antifungals. Ultimately, leveraging drug repurposing alongside nanotechnology may accelerate the translation of statin‐based antifungal therapies into clinical practice, providing an innovative and cost‐effective avenue to broaden the therapeutic arsenal against mucosal *Candida* infections.

## 1 Introduction

Fungal infections remain a pressing global health challenge, particularly among immunocompromised populations [1]. Members of the genus *Candida* account for approximately 80% of all fungal infections worldwide and represent a leading cause of opportunistic mycoses [2, 3]. While *Candida albicans* is the most frequently isolated species, non‐*albicans* species, including *Candida glabrata* , *Candida parapsilosis* , *Candida tropicalis* , *Candida krusei* , and the emerging multidrug‐resistant *Candida auris* , are gaining clinical significance due to rising resistance to frontline antifungal classes [4, 5]. Reflecting this growing concern, in 2022 the World Health Organization designated *C. albicans* as one of only four fungal pathogens classified as a “critical threat” to public health [6]. Clinically, *C. albicans* infections, collectively referred to as candidiasis, can manifest as mucosal infections affecting the oral cavity, esophagus, or genital tract, with vulvovaginal candidiasis (VVC) ranking among the most prevalent mucosal forms. VVC affects an estimated 138 million women annually worldwide and is characterized by a high recurrence rate, substantially impairing quality of life [7, 8].

The standard management of mucosal candidiasis relies primarily on azole antifungals, with polyenes used less frequently and typically administered either orally or topically [9]. However, these therapeutic options are constrained by the limited number of available antifungal agents, treatment‐associated adverse effects, and the escalating problem of drug resistance [10, 11]. The rise in antifungal resistance, driven in part by empirical prescribing practices, underscores the urgent need for innovative therapeutic strategies and strengthened antifungal stewardship [12, 13]. Consequently, the development of alternative interventions has become a global health priority. Yet, the conventional pathway for novel drug discovery, development, and regulatory approval can span approximately 15 years, rendering it inadequate to address the immediate and growing clinical demands posed by resistant *Candida* infections [14, 15].

Within this context, drug repurposing has emerged as a compelling and pragmatic strategy, leveraging agents with well‐established safety and efficacy profiles to shorten development timelines and reduce costs [16]. Among the pharmacological classes under investigation, statins, widely prescribed for the management of hypercholesterolemia, have demonstrated notable antifungal potential and are being repurposed for the treatment of candidiasis [17]. Statins act as inhibitors of hydroxymethylglutaryl‐CoA (HMG‐CoA) reductase, and their antimicrobial activity is thought to stem from the inhibition of isoprenoid biosynthesis, thereby disrupting key processes in fungal physiology, including ergosterol biosynthesis, modulation of G‐protein signaling, and activation of Ras‐dependent pathways [18, 19].

Another promising avenue to address the current therapeutic limitations is the development of advanced pharmaceutical formulations capable of improving the delivery and performance of antifungal agents. Formulation strategies such as polymeric films [20] and in situ gel [21] can improve local drug concentrations and sustain therapeutic activity. More broadly, advanced drug delivery platforms can improve solubility [22], stability [23], bioavailability [24], and site‐specific targeting [25], ultimately increasing treatment efficacy while minimizing systemic exposure. Among these, nanotechnology‐based approaches have transformed the field by enabling the engineering of nanoscale carriers capable of entrapping, encapsulating, dissolving, or binding active pharmaceutical ingredients (APIs) within their matrix [26].

A wide range of nanosystems has been investigated, including lipid‐based nanoparticles, such as liposomes, solid lipid nanoparticles (SLNs) and nanostructured lipid carriers (NLCs), as well as polymeric nanoparticles [27]. Liposomes, composed of phospholipid bilayers surrounding an aqueous core, can encapsulate both hydrophilic and hydrophobic molecules while offering biocompatibility, biodegradability, and protection against enzymatic degradation [28]. SLNs, characterized by a solid lipid core stabilized with surfactants, provide high stability, reproducibility, and scalability, whereas NLCs, incorporating both solid and liquid lipids, improve drug loading, stability, and release control [29].Polymeric nanoparticles, such as those fabricated from polylactic‐co‐glycolic acid (PLGA), enable controlled and sustained drug release, with architectures that can be tuned as nanospheres or nanocapsules depending on polymer arrangement [30]. Particularly relevant for mucosal delivery, chitosan‐based nanoparticles, derived from the deacetylation of chitin, combine low toxicity, biocompatibility, and intrinsic antimicrobial properties with strong mucoadhesive capacity, thereby prolonging retention at the site of infection and facilitating localized drug release [31].

Building on this context, the present review examines innovative strategies for the treatment of mucosal candidiasis, with particular emphasis on drug repurposing and the potential of statins as antifungal agents. Furthermore, it discusses advanced formulations and nanotechnology‐driven delivery systems as approaches to enhance the therapeutic performance of statins in this setting, drawing on recent evidence from the literature.

### 1.1 C. albicans

The Candida genus is the primary group of yeasts responsible for causing opportunistic infections in humans [32]. Approximately 200 Candida species are documented in the literature, with 20 known to cause infections in humans [33, 34]. The main opportunistic species found in healthy individuals include *C. albicans* , *C. glabrata* , *C. tropicalis* , *C. parapsilosis* , and *C. krusei* [35]. These species can be present symbiotically in the oral, gastrointestinal, and urogenital microbiota of 50%–70% of healthy people [36, 37].

Among Candida species, *C. albicans* stands out as the primary opportunistic pathogen. This dimorphic fungus transitions from yeast to hyphal form under pathogenic conditions, particularly in immunocompromised hosts [38]. This polymorphic species exhibits significant versatility in transforming from a harmless commensal microorganism into a pathogen due to its virulence factors, making it one of the most common species causing candidiasis [39]. The primary virulence factors of *C. albicans* include adherence to host cells, morphogenesis, and the secretion of hydrolytic enzymes that damage tissues [40]. Morphogenesis, in particular, is crucial for pathogenicity, as *C. albicans* adheres to host cells as yeast and invades tissues as hyphae [41]. Risk factors such as diabetes mellitus can enhance the action of these virulence factors. Also, elevated blood glucose levels activate enzymes like aspartyl protease (Sap), phospholipases, and candidalysin, which destabilize cell membranes, promoting cell lysis and tissue penetration [42].

Factors such as a limited antifungal spectrum, irrational use of antimicrobials, and prolonged ICU stays are correlated with an increase in invasive candidiasis and fungal resistance [43]. Fungal resistance in *C. albicans* is multifaceted, involving several mechanisms such as biofilm formation, mitotic mutations, and genetic recombination. Biofilms act as protective barriers, significantly enhancing fungal resistance by shielding *Candida* species from conventional antifungal agents [44, 45]. Furthermore, mutations in the *ERG11* gene, which encodes the essential enzyme 14α‐demethylase (Erg11p) for ergosterol biosynthesis, along with overexpression of ergosterol in the plasma membrane, further contribute to antifungal resistance [2, 46].

Moreover, mutations in the ERG11 gene reduce the affinity of Erg11p for fluconazole, complicating the treatment of candidiasis [47, 48]. Moreover, the activation of efflux pumps and loss‐of‐function mutations in the ERG3 gene, which encodes the enzyme C‐5 sterol desaturase, lead to ergosterol depletion and the accumulation of alternative sterols. These changes contribute to cross‐resistance to both azoles and polyenes [49].

## 2 VVC

VVC is an acute inflammatory condition caused by the overgrowth of Candida species, significantly impacting the quality of life for many women of reproductive age [50, 51]. Key symptoms include pruritus, abnormal vaginal discharge, dyspareunia, and external dysuria [52]. Factors that can induce VVC encompass immunosuppression, genetic polymorphisms, allergies, use of antibiotics and glucocorticoids, pregnancy, stress, sexual activity, and uncontrolled diabetes [53, 54].

VVC is generally classified into uncomplicated and complicated cases. Uncomplicated VVC presents as mild, sporadic infections with moderate symptoms. In contrast, complicated cases, such as recurrent vulvovaginal candidiasis (RVVC), VVC during pregnancy, or VVC associated with underlying conditions like immunosuppression and diabetes, are more severe. It is estimated that 10%–20% of women experience complications requiring specialized diagnostic and therapeutic approaches [55, 56]. Differentiating between VVC and RVVC is crucial for selecting the most effective treatment [57]. RVVC is defined as three or more symptomatic episodes within 12 months, with *C. albicans* being the causative agent in at least three‐quarters of these cases [58].

The diagnosis of VVC typically involves clinical examination, microscopy of vaginal fluid, and mycological culture [59]. Observing budding yeasts, hyphae, or pseudohyphae is crucial for confirming VVC [60]. While mycological culture is considered the gold standard for diagnosis, its analysis time of 48–72 h can delay results, making it less practical for rapid diagnosis [61]. Consequently, diagnosis often relies on clinical symptoms, which can be imprecise and have low specificity, leading to a 50% rate of diagnostic imprecision [62]. This empirical approach can result in inappropriate antifungal prescriptions, contributing to increased resistance and recurrent [63].

**FIGURE 1:**
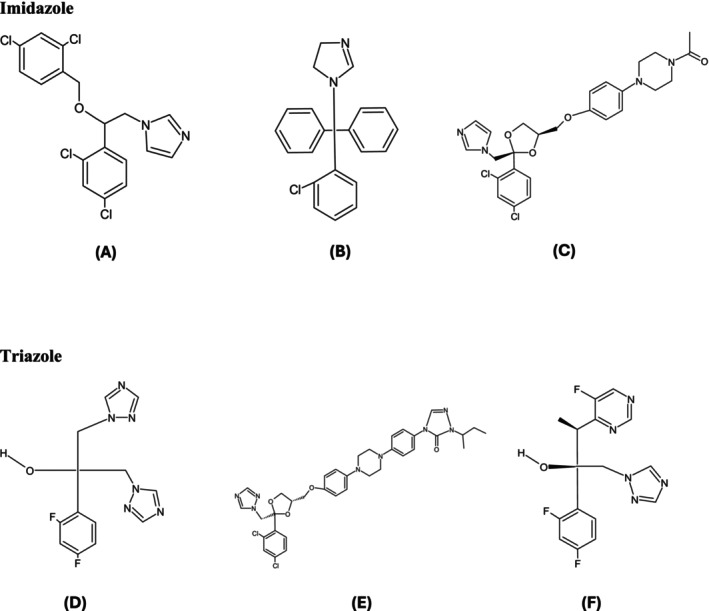
Chemical structures of imidazole and triazoles class. (A) Miconazole, (B) clotrimazole, (C) ketoconazole, (D) fluconazole, (E) itraconazole, and (F) voriconazole. Structures designed by ChemSketch.

Treatment of VVC can involve topical, oral, or combined therapies depending on whether the case is complicated or uncomplicated [56]. Azole antifungals (Figure 1) are the first‐line treatment options [64]. Topical azoles include clotrimazole, miconazole, tioconazole, butoconazole, and terconazole, while oral options approved by the FDA are fluconazole, itraconazole, and oteseconazole [65]. These drugs act by inhibiting lanosterol 14‐alpha demethylase, a key enzyme in ergosterol synthesis, which is essential for fungal cell wall formation [66]. However, orally administered azoles can have adverse effects, including neutropenia, thrombocytopenia, hepatotoxicity, gastrointestinal disorders, and neurological disorders [67]. Fluconazole, the most commonly used drug, has limitations such as contraindication during pregnancy, in vitro resistance in *C. albicans* , and potential to promote resistance in non‐*albicans* species, leading to further complications [68].

**FIGURE 2:**
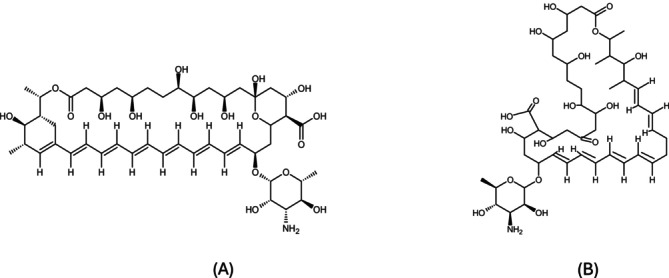
Chemical structures of polyenes class. (A) Amphotericin B and (B) nystatin. Structures designed by ChemSketch.

Another class of antifungal agents used in the treatment of VVC is polyenes, including amphotericin B and nystatin (Figure 2). Polyenes exert fungistatic and fungicidal effects by increasing the permeability of the fungal cell membrane [69]. They achieve this by binding to ergosterol, which disrupts membrane integrity and causes the leakage of essential ions, such as sodium and potassium, as well as sugars, leading to fungal cell death [70].

Although various treatments are available, managing VVC remains challenging, primarily due to the growing resistance of *Candida* species to commonly used antifungal agents. Notably, recent studies report a rising prevalence of resistance, particularly against azole antifungals like fluconazole, widely regarded as a first‐line therapy [70]. Furthermore, non‐*albicans Candida* species, which are often less responsive to azoles, have been increasingly implicated in VVC cases. This shift in fungal epidemiology is exacerbated by the overuse and misuse of antifungals, contributing to a broader resistance problem [71] Recent findings also underscore the emergence of multidrug‐resistant strains, making effective treatment even more difficult [6]. The increasing trends in antifungal resistance underscore the urgent need for innovative therapeutic approaches and improved stewardship practices to enhance the management of VVC and curb the progression of resistance [72].

## 3 Oral Candidiasis (OC) [33, 34]

OC, also known as oropharyngeal candidiasis, is a fungal infection caused by Candida species that affects the oral cavity [73]. The primary opportunistic pathogen responsible for OC is *C. albicans* , accounting for approximately 95% of diagnosed cases. This prevalence is due to *C. albicans* being a commensal organism in the oral cavity of healthy individuals, with around 80% of the population being asymptomatic carriers [74]. Commonly referred to as thrush, OC is characterized by symptoms such as dysphagia, pain, and changes in taste, and can affect various parts of the oral and buccal mucosa, including the palate, gums, tongue, and oropharynx [75].

OC presents in several clinical forms: acute candidiasis (erythematous and pseudomembranous types), chronic candidiasis (erythematous and hyperplastic types), and angular cheilitis (linear gingival erythema and median rhomboid glossitis) [76, 77]. Pseudomembranous candidiasis is marked by white, creamy plaques that are difficult to remove and cause a burning sensation. Erythematous candidiasis features red patches with burning sensations, hyperplastic candidiasis presents as asymptomatic white plaques, and angular cheilitis is characterized by fissured red patches with sores [78].

Diagnosis of OC involves a comprehensive clinical examination and microbiological investigations. Laboratory tests typically include histology with periodic acid–Schiff (PAS) staining and polymerase chain reaction (PCR). PAS staining helps identify *C. albicans* hyphae, providing confirmation of the infection [79, 80].

Although OC has a low lethality rate, if left untreated, it can progress to a chronic form and potentially lead to systemic candidiasis [81]. Treatment usually involves topical antifungal agents due to their effectiveness and minimal adverse effects [82]. The most commonly used topical antifungals for initial treatment include miconazole, clotrimazole, and nystatin, with nystatin being the most frequently prescribed [83]. For patients intolerant to or inadequately responsive to topical treatments, oral antifungals such as fluconazole, itraconazole, and miconazole are recommended [84].

## 4 Esophageal Candidiasis (EPC)

EPC is an infectious disease that primarily affects patients with chronic illnesses and those who are immunosuppressed, such as individuals with human immunodeficiency virus (HIV), organ transplant recipients, or those undergoing immunosuppressive therapy. It targets the esophagus, which is the second most susceptible site for infection in the gastrointestinal tract, following the oropharynx [85]. Patients undergoing cancer treatment are also at increased risk of EPC due to factors such as neutropenia, mucosal lesions, antibiotic use, and prolonged corticosteroid therapy [86].

EPC is predominantly caused by *C. albicans* and is more frequently observed in patients with carcinoma compared to those with esophagitis [87]. The clinical manifestations include dysphagia, odynophagia, dyspepsia, nausea, and vomiting [88]. Diagnosis is typically straightforward, with endoscopy being the primary diagnostic procedure. *C. albicans* hyphae can be visualized in the squamous epithelium using hematoxylin–eosin staining. For more precise confirmation, special stains such as Gomori Methenamine Silver and Periodic Acid‐Schiff can be employed [89].

Treatment options include both oral and intravenous antifungal therapies. Oral fluconazole, at a dosage of 200–400 mg daily for 14–21 days, is the most commonly prescribed treatment [85].

## 5 Drug Repurposing Strategies

The development of new antifungal drugs targeting resistance mechanisms has become a global necessity. However, the time required to develop and register new drugs falls short of meeting current demands [15]. The drug development process is time‐intensive and costly, comprising four key stages: drug discovery and synthesis, preclinical studies, clinical trials, and post‐marketing safety monitoring. Completing these stages can take approximately 12–17 years [14].

In this context, drug repurposing presents a promising solution to current challenges. Also known as repurposing, this strategy involves identifying new therapeutic uses for an existing API that is already marketed for its primary therapeutic action [90]. One of the main goals of drug repurposing is to shorten the research timeline for discovering new molecules by approximately 50%–60% by utilizing chemically well‐characterized and biologically safe molecules [91]. Pharmaceutical companies support this approach as it can reduce costs and optimize profit margins [92].

Currently, computational chemistry and bioinformatics are essential tools for enabling in silico screening of chemical structures and identifying potential new therapeutic targets. These tools facilitate the selection of substances for subsequent in vitro and in vivo repurposing tests [93]. Key methodologies include (1) structure‐based approaches such as molecular docking, structural similarity of binding sites, and receptor‐based pharmacophores; (2) expression‐based approaches including drug‐disease and drug–drug network expression profiles and similarities; and (3) ligand‐based approaches like similarity searches, side effect similarities, QSAR, and machine learning [94].

In the literature, several examples of successful drug repurposing can be highlighted: sildenafil, originally researched as an antihypertensive, was repurposed for erectile dysfunction [95]. Metformin, used for treating diabetes mellitus, has been repurposed for colorectal, prostate, ovarian, and breast cancer [96, 97]. Additionally, Ferreira et al. [98] suggested the potential repurposing of the anti‐inflammatory drug indomethacin for melanoma, based on published data.

The repurposing of drugs for antimicrobial use is of particular interest due to increasing fungal and bacterial resistance, as well as the recent COVID‐19 pandemic, which has spurred the scientific community to explore potential treatments for future pandemics [99, 100]. Ribeiro et al. [101] describe the repurposing of atorvastatin as an anticryptococcal agent. Blaskovich et al. [102] reported the repurposing of cannabidiol, known for its antiepileptic potential, against gram‐negative bacilli (GNB), where it demonstrated efficacy in inhibiting and eradicating biofilms.

The strategy of repurposing drugs for treating fungal diseases is well‐documented in the literature and is gaining increasing attention within the scientific community. A study by Lei et al. [103] evaluated the antifungal activity of vitamin D3 (VD3) against various *Candida* species. In vitro assays confirmed that VD3 inhibited the growth of *Candida* spp. in a broad‐spectrum, dose‐dependent manner and demonstrated a significant antifungal effect during the biofilm formation stages of *C. albicans* .

Tricyclic antidepressants and Selective Serotonin Reuptake Inhibitors (SSRIs) are also recognized in the literature for their antifungal properties [104]. A study involving three tricyclic antidepressants, doxepin, imipramine, and nortriptyline, demonstrated through in vitro assays that this class of drugs can inhibit hyphae and biofilm formation and also kill cells within mature biofilms in *C. albicans* species [44]. Regarding SSRIs, paroxetine, fluoxetine, and sertraline were tested against fluconazole‐resistant *Candida* spp. These drugs exhibited a wide range of minimum inhibitory concentrations (MICs): 20–160 μg/mL for fluoxetine, 10–20 μg/mL for sertraline, and 10–100.8 μg/mL for paroxetine. They induced fungal apoptosis by damaging the mitochondrial membrane, with fluoxetine notably reducing the mature biofilm in all tested species [105].

SSRIs have been documented in the literature both as standalone treatments and in combination with conventional antifungal drugs like fluconazole to explore synergistic effects. For instance, fluoxetine combined with fluconazole was tested on 29 *Candida* strains isolated from patients with VVC, showing synergistic activity against six strains. The MIC of fluconazole for resistant strains decreased by up to 64‐fold when the drugs were used in combination [106]. Additionally, studies have explored azole synergism with sertraline. Rodrigues et al. [45], evaluated the association of sertraline with fluconazole, itraconazole, and amphotericin B in both planktonic and biofilm cells. Sertraline demonstrated a fungicidal effect, showing 93.75% synergy with fluconazole, 37.5% additive effects, and 25% synergy with itraconazole. Additionally, it exhibited 43.75% additive effects with amphotericin B. Furthermore, sertraline inhibited 85% of biofilm formation and maturation, with all effective concentrations remaining within safe ranges.

## 6 Repurposing Statins for Antifungal Therapy

**FIGURE 3:**
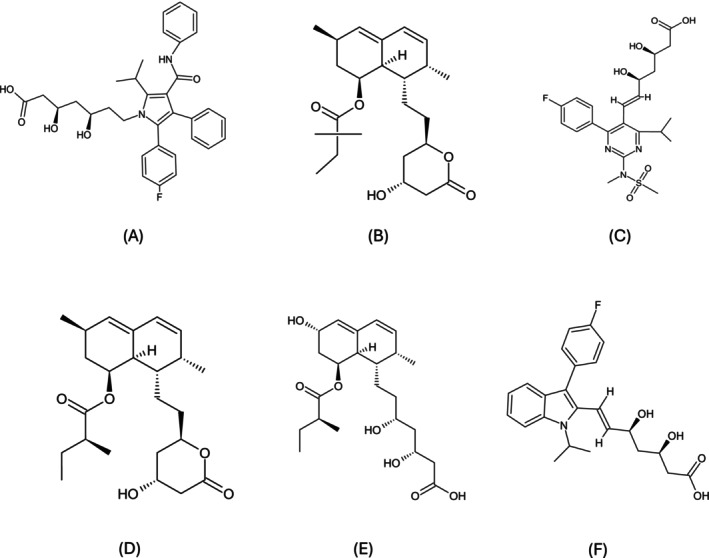
Chemical structures of statins. (A) Atorvastatin, (B) simvastatin, (C) rosuvastatin, (D) lovastatin, (E) pravastatin, and (F) fluvastatin. Structures designed by ChemSketch.

Statins, first discovered in the 1970s, have become essential in clinical practice for managing hyperlipidemia and reducing cardiovascular risk [107]. Commonly used statins include atorvastatin, simvastatin, rosuvastatin, lovastatin, pravastatin, and fluvastatin (Figure 3). These drugs function by inhibiting hydroxymethylglutaryl coenzyme A (HMG‐CoA) reductase, a crucial enzyme in cholesterol synthesis. In addition to their primary role in lowering LDL cholesterol, statins have demonstrated potential in various off‐label applications, such as anti‐inflammatory and immunomodulatory effects, which continue to drive research into their expanded therapeutic uses [108, 109].

**FIGURE 4:**
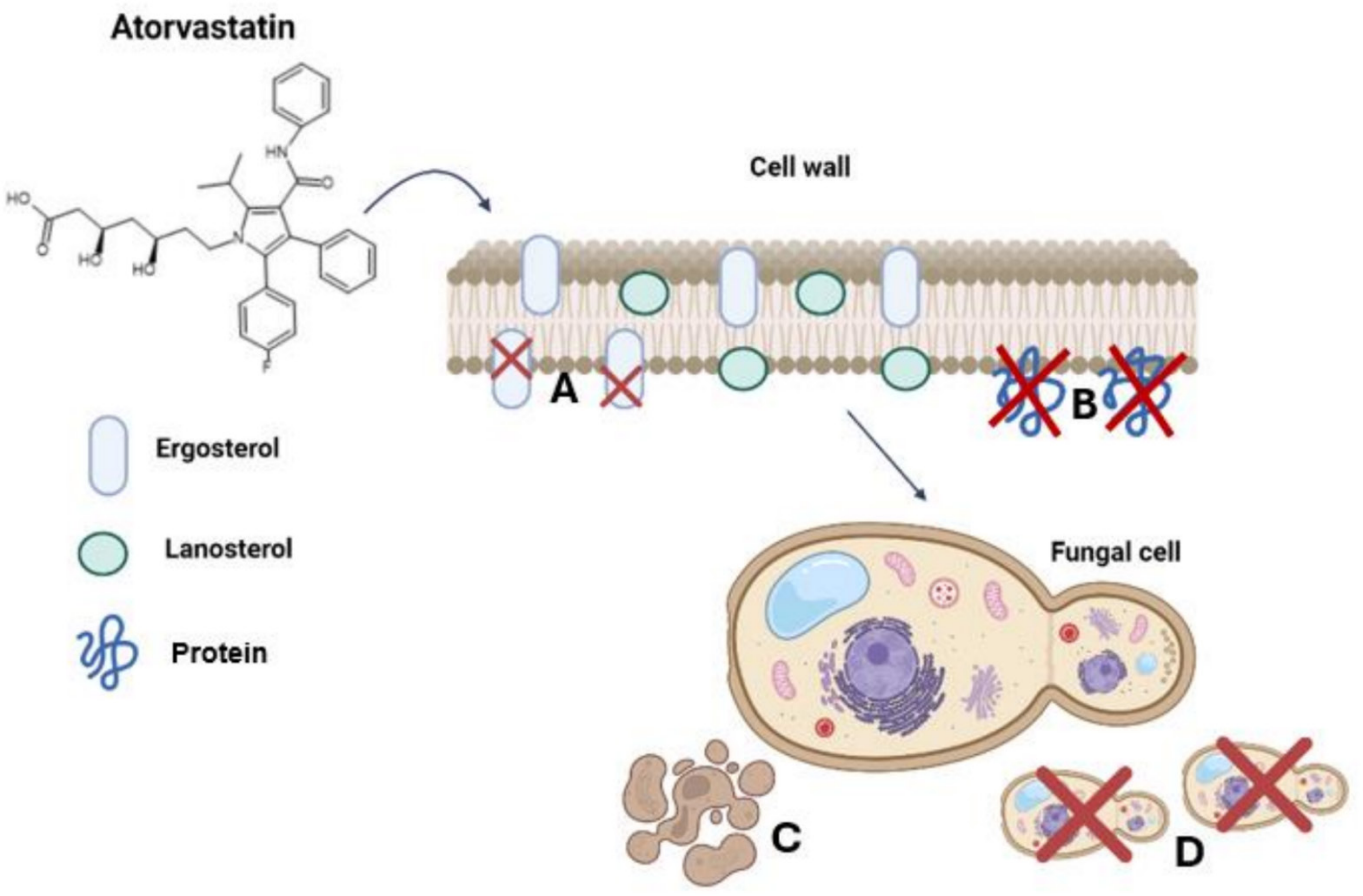
Potential mechanisms of the antifungal effect of ATV. (A) Inhibition of ergosterol synthesis by HMG‐CoA reductase present in the fungal cell wall; (B) prenylation of proteins and disruption of function; (C) mitocondrial disruption and apoptosis by oxidative stress; (D) morphogenesis and mating disruption by HMG‐CoA reductase. Created using BioRender.com.

Moreover, statins exhibit a broad spectrum of biological activities, including antimicrobial, antiparasitic, antibacterial, antiviral, and antifungal potential [110, 111, 112, 113]. Although the exact mechanism of their antifungal action remains unclear, studies suggest that statins may exert their effects through various pathways. These include inhibiting ergosterol synthesis in fungal cells by blocking HMG‐CoA reductase, interfering with protein prenylation and function, disrupting mitochondrial function and inducing apoptosis, and affecting fungal cell morphogenesis and reproduction [114]. Figure 4 illustrates the potential mechanisms of antifungal action of atorvastatin (ATV). Studies suggest that this statin may exert its antifungal effects through various mechanisms, contributing to its significant activity against different *Candida* strains, particularly *C. albicans* [115].

By inhibiting HMG‐CoA reductase, statins lead to a reduction in the levels of mevalonate and farnesyl pyrophosphate in fungal cells, thereby influencing various processes regulated by these compounds. This includes the inhibition of isoprenylation, which affects the translation of proteins related to the regulation of cell growth, specialization, and programmed cell death, as well as negatively impacting the activation of essential cellular proteins, such as those involved in respiration and iron metabolism [116, 117].

Extensive studies suggest that statins may impact not only ergosterol synthesis but also other cellular targets, including mitochondrial function and the apoptosis cascade. Ajdidi et al. [118] demonstrated that statins inhibit the growth of clinically relevant fungi such as *Aspergillus fumigatus* and *C. albicans* . Furthermore, the study reported that prolonged exposure of *C. glabrata* cells to statins increases the proportion of small mutations, suggesting a potential effect on mitochondrial DNA integrity. Additionally, Roze et al. [119] found that lovastatin induces cell death resembling apoptosis in *Mucor racemosus*. Lovastatin halts sporangiospore germination, reduces cell growth, and causes loss of viability characterized by cell shrinkage, increased density, and cytoplasmic condensation. It also triggers DNA fragmentation, resulting in the formation of nucleosomes and multimers.

Bellanger et al. [120] investigated the effects of lovastatin, atorvastatin, and simvastatin on *Rhizopus oryzae* and observed DNA fragmentation and a reduction in spore germination. They also reported that exposure to lovastatin resulted in macroscopic melanin loss and diminished virulence in mucormycosis models using flies and mice. Similarly, Bocate et al. [121] demonstrated that simvastatin effectively controlled the growth of toxigenic *Aspergillus* species, reducing biofilm production and indicating potential as an antifungal agent. Additionally, statins may stimulate farnesol‐dependent pathogenicity factors, such as the transition from yeast to hyphae and biofilm formation, which are key resistance mechanisms in fungi [115].

Focusing specifically on the antifungal activity of statins against candidiasis, Esfahani et al. [122] observed that ATV was effective against all tested *C. albicans* strains, while these strains were resistant to fluconazole and nystatin, underscoring ATV's potential as an antifungal agent. ATV also demonstrated significant antifungal activity against various Candida species, with notable selectivity for *C. albicans*, including strains resistant to fluconazole, ketoconazole, itraconazole, and voriconazole, with minimum inhibitory concentrations ranging from 16 to 32 μg/mL [19]. Ajdidi et al. [118] evaluated the antifungal efficacy of ATV both in vitro and in vivo, finding that ATV reduced *C. albicans* growth by 74.6% within 24 h. In vivo studies using *Galleria mellonella* larvae infected with *C. albicans* demonstrated that ATV improved larval survival. Additionally, De Oliveira Neto et al. [9] tested ATV in a VVC model with Wistar rats and reported that emulgels containing 3% ATV reduced the fungal load by 65% after 9 days of treatment.

To date, the antifungal activity of ATV has been demonstrated predominantly in in vitro studies. More recently, however, a pioneering clinical investigation assessed the impact of therapeutic statin use on oral *Candida* colonization in hyperlipidemic patients [123]. This study enrolled 50 patients receiving regular statin therapy (HLS group) and 50 hyperlipidemic patients not on statins (HLNS group), matched for sex, age, and dental status. The prevalence and colony counts of *Candida* were significantly lower in the HLS group compared with the HLNS group (20 patients, 40% vs. 30 patients, 60%, respectively; *p* = 0.040). These findings suggest that statin therapy may be associated with a reduction in both the prevalence and burden of oral *Candida* colonization. Nevertheless, because this was an exploratory study involving patients already under statin treatment, dosing regimens were not standardized. At enrollment, 42 individuals (84%) were receiving 20 mg daily, while smaller proportions were on 10 (6%), 40 (6%), 60 (2%), or 80 mg (2%). This heterogeneity highlights the need for larger, well‐controlled trials to clarify the relationship between the antifungal effects of statins and variables such as dose, statin type, and duration of therapy, as well as to establish their clinical efficacy in managing oral *Candida* infections [123].

Complementing this preliminary clinical evidence, in vitro studies with clinical isolates further support the antifungal potential of statins. For instance, Golestannejad et al. [124] compared the antifungal activity of fluconazole and ATV against *Candida* spp. isolated from patients undergoing head and neck radiotherapy, a condition frequently associated with refractory OC. Oral swabs from 33 patients were collected before and 2 weeks after radiotherapy, and antifungal susceptibility was evaluated using minimum inhibitory concentration (MIC) and minimum fungicidal concentration (MFC) assays. While ATV exhibited measurable antifungal effects, fluconazole demonstrated significantly greater potency at substantially lower concentrations against *C. albicans* , *C. tropicalis* , and *C. glabrata* , both before and after radiotherapy. These results reinforce the antifungal potential of ATV while emphasizing the need to explore synergistic combinations to enhance its efficacy, thereby supporting its continued evaluation in clinical settings.

To further enhance this antifungal potential, several strategies have been proposed to enhance the antifungal efficacy of statins while minimizing required dosages. One approach is pharmacological synergism with established antifungal agents, particularly azoles [116]. For instance, itraconazole and rosuvastatin combinations have produced marked synergy against 11 clinical isolates of *C. albicans* [125]. Likewise, fluconazole and ATV combinations have shown fungicidal activity within 24 h against fluconazole‐resistant *C. albicans* , significantly inhibiting biofilm maturation and filamentation [126]. ATV has also demonstrated broad synergistic interactions with fluconazole, itraconazole, voriconazole, and ketoconazole against multidrug‐resistant *C. albicans* , suggesting superior potential relative to other statins [115]. While these studies did not consistently assess resistance profiles, the observed synergy raises the possibility that such combinations could be valuable even in certain drug‐resistant contexts, particularly when resistance mechanisms do not preclude the complementary actions of statins on fungal membrane integrity and signaling pathways [127].

Beyond combination therapy, advanced drug delivery systems represent another promising strategy. Nanoparticle‐based carriers such as lipid nanoparticles, polymeric nanocapsules, and liposomes can enhance ATV's solubility, stability, and targeted delivery to infection sites, thereby improving therapeutic efficacy and reducing systemic exposure [27]. This is particularly relevant given that statins exhibit poor aqueous solubility and chemical instability, factors that can limit drug availability at the site of infection and compromise biological activity [128, 129]. Complementary formulation strategies, such as mucoadhesive hydrogels, biofilm‐disrupting adjuvants, and stimulus‐responsive release systems, may further potentiate antifungal effects and improve clinical translation [130].

## 7 Innovative Drug Delivery Systems for Statins: Leveraging Nanotechnology and New Formulations in the Treatment of Mucosal Candidiasis

Nanotechnology plays a pivotal role in advancing drug delivery systems and has reshaped the pharmaceutical landscape by enabling innovative approaches to disease management [131]. Numerous studies have demonstrated the successful encapsulation of statins in diverse nanoparticle platforms, reporting marked improvements in stability, reduced toxicity, controlled release, and enhanced therapeutic efficacy across various pathologies. Encapsulation not only protects statins from chemical and enzymatic degradation but also facilitates sustained and targeted delivery, thereby optimizing drug concentration at the intended site of action. Such delivery strategies have been associated with increased bioavailability and reduced adverse effects compared with conventional statin formulations [27, 132].

Within this framework, recent studies indicate that the nanostructuring of antifungal drugs can significantly enhance their efficacy, particularly against resistant strains of *Candida* [133]. This innovative approach improves drug solubility and bioavailability, allowing for more effective targeting of fungal cells. The nanoscale formulations can facilitate better penetration into biofilms, which are often associated with chronic infections and resistance mechanisms [134]. Additionally, nanostructured antifungals may reduce toxicity and side effects, enabling higher doses to be administered safely. Overall, these advancements in drug delivery systems hold great promise for improving treatment outcomes against challenging fungal infections, especially those caused by resistant Candida strains [135, 136].

This opens up promising opportunities for further investigation and investment in this potential avenue, which could lead to novel therapeutic strategies that leverage the antifungal properties of statins, particularly in the context of resistant strains of fungi [137]. Integrating nanotechnology with statin therapy represents a compelling direction for addressing the growing challenge of antifungal resistance, and further exploration could yield valuable insights for enhancing treatment outcomes in patients suffering from fungal infections. However, there is a notable scarcity of published studies on this topic, indicating a significant gap in the current research landscape [138].

In an interesting study, ATV was encapsulated in emulsomes (ATO‐EMLs) aiming to enhance its topical antifungal activity. The nanoparticles exhibited a monodisperse distribution, a size of 250 nm, a zeta potential of approximately −25 mV, and an encapsulation efficiency exceeding 80%. The authors observed that emulsomes provided controlled release of ATV, with 75% of the drug released after 72 h. The formulation showed no signs of skin irritation in an in vivo assay conducted on albino rats. Additionally, in vitro microbiological assays demonstrated that nanoencapsulated ATV reduced the minimum inhibitory concentration against *C. albicans* compared to the free drug. Furthermore, the antifungal activity was similar to that of the commercial product (clotrimazole 1%). Notably, the in vivo antifungal activity of ATO‐EMLs, evaluated in albino rats infected with *C. albicans* , was significantly greater than in untreated animals. The authors argue that the results are promising, aiming to provide a therapeutic alternative for the treatment of fungal infections, particularly in cases of species resistant to conventional drugs [137].

Likewise, Nour et al. [138] proposed the development of mucoadhesive films obtained by 3D printing containing ATV‐loaded liposomes (ATV‐Lip) for the treatment of OC. ATV‐Lip exhibited suitable characteristics in terms of size (223.3 ± 2.1 nm), polydispersity index (0.12 ± 0.001), zeta potential (−18.2 ± 0.3 mV), and encapsulation efficiency (81.15 ± 1.88%), and provided controlled drug release over 24 h. Additionally, the nanoencapsulated ATV did not reduce the viability of gingival fibroblasts, suggesting it is safe for its intended use. The authors confirmed the mucoadhesive capability and controlled release profile of ATV (79.4 ± 1.4% over 24 h) in the developed films. In vitro antifungal activity of the ATV‐Lip against fluconazole‐resistant *C. albicans* was superior to free ATV in the time‐kill assay. However, in the determination of the minimum inhibitory concentration and in the agar diffusion assay, better results were reported for the free ATV. In the specific case of the diffusion assay, a similar behavior was observed with the films. The authors argue that this finding may be related to the controlled release of ATV and its difficulty of diffusion from the film polymeric matrix. Despite this, in vivo assays using a rabbit OC model demonstrated enhanced antifungal activity and a significant reduction in inflammation for the films compared to other treatment groups.

A recent study by Molania et al. [139] developed SLNs incorporating ATV for the treatment of denture stomatitis caused by *C. albicans* . Glyceryl monostearate served as the solid lipid, while Tween 80 and Span 80 were employed as surfactants. The resulting SLNs exhibited particle sizes ranging from 188.93 to 236.2 nm, zeta potential between −20.4 and −27 mV, and a high encapsulation efficiency of 85.88%. Antifungal susceptibility testing showed that nanoencapsulation markedly reduced the MIC of ATV against *C. albicans* to 0.015–0.5 μg/mL, compared with 1–32 μg/mL for the free drug. In vitro release studies revealed a controlled release profile, with 47% of the payload released within 6 h and 80% within 24 h. Collectively, these findings support AT‐SLNs as a promising platform for antifungal therapy in denture stomatitis.

Beyond current examples with statins, formulation strategies developed for other antifungal agents provide valuable proof‐of‐concept approaches that could be adapted to enhance statin delivery for mucosal candidiasis. In this sense, nanotechnology and innovative formulation strategies have also been employed to address fungal resistance mechanisms, offering concepts that could be extrapolated to statin‐based antifungal therapies [21]. Wang et al. [140] designed a multifunctional “three‐in‐one” thermosensitive vaginal gel combining mucoadhesive properties, mucus‐barrier penetration, and biofilm‐disrupting activity. The poloxamer‐based gel incorporated two nanoparticle systems: anti‐biofilm polymeric nanoparticles (AFNPs) co‐loaded with farnesol (FAR) and amphotericin B (AMB), which synergistically inhibited *C. albicans* biofilms; and mucus‐penetrating polymeric nanoparticles (BP NPs) containing bromelain (BRO), which facilitated AFNPs transport through the mucosal barrier. This platform demonstrated prolonged vaginal adhesion, resistance to self‐clearing, and controlled release, leading to enhanced antifungal efficacy in vitro and in vivo, as well as reduced inflammation in murine models of VVC. Notably, the gel exhibited a sol–gel transition near body temperature and reduced mucus viscosity, further promoting local penetration features that could be leveraged in statin delivery systems targeting mucosal infections.

Similarly, Kraisit et al. [141] developed chitosan‐based oral films via hot homogenization, incorporating SLNs composed of glyceryl monostearate and Tween 80 loaded with fluconazole (FZ) for the treatment of OC. A Box–Behnken design was used to optimize the formulation, yielding FZ‐SLNs with particle sizes between 32.86 and 269.3 nm. Permeation studies using porcine buccal mucosa showed greater fluconazole permeation from chitosan films containing SLNs compared with those containing free drug. In vitro antifungal assays against *C. albicans* confirmed improved activity for the nanoformulated films, as evidenced by larger inhibition zones. Such a mucoadhesive, nanoparticle‐enhanced platform could be adapted to statins to improve mucosal retention, permeation, and antifungal potency.

## 8 Conclusion

Given the growing challenge of antifungal resistance in the treatment of candidiasis, drug repurposing emerges as a promising and innovative strategy capable of accelerating the introduction of new therapeutic alternatives. In this context, statins, widely recognized for their lipid‐lowering effects, have demonstrated significant antifungal potential in recent studies. Among them, atorvastatin stands out for its consistent in vitro and in vivo performance, suggesting applicability not only as an alternative therapeutic agent but also as a viable option for combination regimens targeting resistant *Candida* spp. infections. Other statins have also shown encouraging antifungal profiles, broadening the therapeutic landscape and warranting further mechanistic and translational research to define their optimal use.

In parallel, the development of advanced drug delivery systems, particularly nanoparticle‐based platforms, offers an important technological pathway to overcome physicochemical limitations, enhance local drug bioavailability, prolong mucosal retention, and minimize systemic exposure. These innovations are especially relevant for topical applications in mucosal candidiasis, where site‐specific delivery and sustained release can significantly improve therapeutic outcomes.

Despite promising preclinical data, the clinical translation of statin‐based antifungal therapies remains underexplored. Notably, no studies to date have specifically investigated vaginal formulations of statins, representing a critical gap in the field. Addressing this gap through well‐designed preclinical models and early‐phase clinical trials could accelerate the repurposing of statins into effective, safe, and accessible options for managing drug‐resistant mucosal candidiasis. Harnessing the dual potential of drug repurposing and nanotechnology could redefine the therapeutic landscape for mucosal candidiasis, transforming statins from cardiovascular agents into precision‐targeted antifungal therapies.

## Author Contributions

Conceptualization: Guilherme Diniz Tavares, Rodrigo Luiz Fabri, Frederico Pittella. Writing – original draft preparation: Dominique Mesquita e Silva, Andrea de Souza Andrioli, Wilson Rodrigues Braz. Writing – review and editing: Dominique Mesquita e Silva, Laís de Souza Lacerda, Lara Melo Campos, Mayara Rodrigues Brandão de Paiva, Rodrigo Luiz Fabri, Frederico Pittella, Guilherme Diniz Tavares. All authors have read and agreed to the published version of the manuscript.

## Conflicts of Interest

The authors declare no conflicts of interest.

## Data Availability

Data sharing not applicable to this article as no datasets were generated or analysed during the current study.
